# Regular Wounding in a Natural System: Bacteria Associated With Reproductive Organs of Bedbugs and Their Quorum Sensing Abilities

**DOI:** 10.3389/fimmu.2017.01855

**Published:** 2017-12-18

**Authors:** Oliver Otti, Peter Deines, Katrin Hammerschmidt, Klaus Reinhardt

**Affiliations:** ^1^Animal and Plant Sciences, University of Sheffield, Sheffield, United Kingdom; ^2^Animal Population Ecology, Animal Ecology I, University of Bayreuth, Bayreuth, Germany; ^3^Zoological Institute, Christian Albrechts University Kiel, Kiel, Germany; ^4^Institute of Microbiology, Christian Albrechts University Kiel, Kiel, Germany; ^5^Applied Zoology, Department of Biology, Technische Universität Dresden, Dresden, Germany

**Keywords:** quorum quenching, interspecific communication, reproductive immunity, genitalia-associated microbes, genital infection

## Abstract

During wounding, tissues are disrupted so that bacteria can easily enter the host and trigger a host response. Both the host response and bacterial communication can occur through quorum sensing (QS) and quorum sensing inhibition (QSI). Here, we characterize the effect of wounding on the host-associated bacterial community of the bed bug. This is a model system where the male is wounding the female during every mating. Whereas several aspects of the microbial involvement during wounding have been previously examined, it is not clear to what extent QS and QSI play a role. We find that the microbiome differs depending on mating and feeding status of female bedbugs and is specific to the location of isolation. Most organs of bedbugs harbor bacteria, which are capable of both QS and QSI signaling. By focusing on the prokaryotic quorum communication system, we provide a baseline for future research in this unique system. We advocate the bedbug system as suitable for studying the effects of bacteria on reproduction and for addressing prokaryote and eukaryote communication during wounding.

## Introduction

All animals live in intimate associations with bacteria, which can live on host surfaces, reside within or between host cells or be associated with specific organ systems (1–3). The entirety of a host and its associated bacterial community (microbiome) is called the metaorganism (4). Studying the effects of microbiomes on host ecology and evolution has become a major line of research (5–8).

Most interactions of the host with bacteria from the environment happen at the host surfaces. One important way by which environmental bacteria might enter the host organism is wounds (9). This is of particular importance in cases where wounding occurs on a regular basis such as during mating (10–12) and when bacteria are transferred to mating partners *via* contaminated reproductive organs. Bacteria are ubiquitously found on male and female genitalia, including insects, birds, or humans (12–16), and copulatory wounding has been shown to be very widespread in the animal kingdom. In many species, males cause micro- and macro-lesions in the female reproductive tract during mating (11) and, even in humans, 10–52% of copulations result in mucous lesions, abrasions, or lacerations of female genital organs [(11) and references therein]. While males may protect their sperm from bacteria, they transfer to females by transferring antimicrobial substances in their seminal fluid alongside the sperm (17, 18), it remains largely unknown how females prepare for bacterial invasions after copulation (19) and how the bacterial community residing in the female responds to the foreign intruders. For example, in other metaorganisms, the resident microbiota plays a critical role in maintaining host health by interacting with invading microbes (8, 20–22).

The host-associated microbial community is shaped by the host but also through interactions within the microbial community. Bacterial communication systems, such as quorum sensing (QS) and quorum sensing inhibition (QSI), influence the stability of the microbial community, and thus the integrity of the metaorganism (23, 24). However, little is known about how these quorum communication systems work between resident and invading microbes.

Quorum sensing and QSI occur within and between bacterial species (25). Essentially, QS regulates the gene expression to produce and release chemical signal molecules called autoinducers in response to fluctuations in bacterial cell-population density (26). These responses include adaptation to the availability of nutrients or the defense against other microorganisms, which may compete for the same nutrients (or hosts). Bacteria also coordinate their behavior in infections with QS, e.g., many pathogenic bacteria coordinate their virulence to evading the immune response of the host and establishing a successful infection.

Competing bacteria species have evolved mechanisms to interfere with each other’s QS communication by quenching the signal molecules, called quorums sensing inhibition (QSI) (25, 27) or by inhibiting each other’s growth (28). As expected, hosts have evolved counteradaptations that interfere with the QS process and limit the spread of information among infecting bacteria, or interfere with bacterial growth to prevent the colonization by bacteria, e.g., through temperature and pH increase (29). Although bacterial communication is currently attracting a lot of interest, not much is known about the distribution of bacteria competent to perform QS, QSI, or growth inhibition in natural bacteria-host systems.

A further important player in the host–microbe interaction has recently been identified. Ismail et al. (30) have shown that the damage of eukaryotic host cells, as occurs during wounding, also releases signals that interfere with bacterial QS systems. This provides, yet another, very fast line of defense once bacteria have bypassed the host’s epithelia. While future work will doubtlessly bring more such exciting research results and will eventually lead to identifying the relative significance of pro- and eukaryotic quorum communication, we here present a first step into that direction. We present a unique arthropod model of regular copulatory wounding—the natural traumatic insemination of bedbugs—and characterize the prokaryotic side of the quorum communication by investigating the ability to perform QS or QSI *in vitro* of bacteria isolated from male and female reproductive organs.

Briefly, the male bedbug possesses a stylet-like copulatory organ (called the paramere) with which it wounds the female (breaches their integument) during every copulation. On the paramere, environmental bacteria have been found (17, 31), which can be transported into the female (17). An experimental overabundance of bacteria on the male’s paramere dramatically accelerated female death and has selected for the evolution of a novel female immune organ (32). This immune organ, the mesospermalege, is filled with immune cells, hemocytes, of more or less unknown function, which significantly reduces the negative effect of wounding and bacterial infection (32). Females have little control over whether or not they mate other than by feeding—fully fed females cannot resist copulation, non-fed females partially can (33). Therefore, fully fed females can expect to be mated, and in order to characterize the prokaryotic quorum communication in our model metaorganism, it is necessary to separate the effects of feeding from the effects of wounding.

The objectives of the current research are: (1) to isolate and identify the site-specific, culturable microbiome of the bedbug, (2) to test the effect of wounding and feeding on the microbiome of female bedbugs, and (3) to quantify the potential for quorum communication of the culturable bacteria species of the bedbug microbiome *in vitro*. We sample eight different bedbug populations for their bacteria using a culture-dependent method. We separately screen the bacteria from the bedbug environment, the cuticle of males and females, the male paramere, the female hemolymph, and mesospermalege. We contrast virgin and mated females to isolate the effect of wounding and feeding on the microbiome and conduct QS and QSI assays to establish the competence of the isolated bacterial lineages for QS, QSI, and growth inhibition. The microbiome varied between mated and non-mated individuals, between fed and non-fed ones as well as between organs. Most of the screened reproductive organs harbored bacteria capable of both signaling pathways, QS and QSI. Our findings provide a baseline for future research in bedbugs and promote it as a system for studying the effects of bacteria on reproduction and prokaryote–eukaryote communication during wounding in a natural system.

## Materials and Methods

### Study Animals

All bedbugs were derived from one large stock population (>1,000 individuals) maintained at the University of Sheffield for more than 6 years (34). We conducted two experiments to (i) obtain all culturable bacteria from specific sites in the female and male bedbug and from the bedbug environment and (ii) disentangle the effect of feeding and mating on bacterial diversity in different sites in the female bedbug. For the bacteria in the bedbug environment, we sampled eight different stocks, six field-caught (five from the UK and one from Kenya) and two long-term lab stocks originally obtained from the London School of Hygiene and Tropical Medicine and from Bayer Environmental Science (Monheim, Germany).

#### Site-Specific, Culturable Microbiome of the Bedbug

To isolate and identify most culturable bacteria from the bedbug microbiome, six different growth media [sterile Grace’s Insect medium (GM; G8142, Sigma-Aldrich, Dorset, UK), NB, NBTA, LB, Potato extract, and R2A] were used to prepare 1.5% agar plates. To assess the diversity of bacteria in the female hemolymph and mesospermalege, we analyzed 3-week old females (*N* = 17), which were either fully mated with randomly picked males from the same stock population (*N* = 12) or left virgin as control (*N* = 5). We allowed the males to copulate for as long as they wanted and removed them immediately after they let go of the female. Thirty minutes after mating, hemolymph samples were taken from all females after which they were dissected to remove the mesospermalege. Additionally, we sampled the microbial diversity on the parameres from five males, which was separated from the rest of the body and incubated in GM before plating.

The microbial diversity in the bedbug environment (stock cultures), and thus possible origins of the microbes on the paramere, the hemolymph, and the mesospermalege was also assessed. To this end, we incubated filter papers from the pots in which the stock populations are kept in 5 ml sterile GM. To ensure high enough bacterial numbers from the tissue samples for detecting them on the different growth media, we incubated all tissues in 250 µl GM for 4 h at 26°C, after which 30 µl were plated using sterile glass beads.

#### The Effect of Wounding and Feeding on the Microbiome of Female Bedbugs

To test whether feeding or mating has an effect on the type or number of different bacteria found in females, we randomly assigned 3-week old females (*N* = 16) to the following four treatments (each *N* = 4): (1) unfed virgins, (2) fed virgins, (3) unfed mated, and (4) fed mated. Females were mated with randomly picked males from our stock population. We allowed the males to copulate for as long as they wanted, measured the mating duration, and removed them immediately after they let go of the female. Fed females mated almost 20 s longer than unfed females (Table 3). Thirty minutes after mating, female bedbugs were sampled for bacteria. We also removed and sampled the paramere of the males (*N* = 7) by incubating it in GM. The males (*N* = 7) and females (*N* = 3) were also rinsed in GM to obtain cuticular bacteria.

### Isolation and Cultivation of Bacteria

Before dissection, females were sterilized using a kimtech tissue dipped in 96% ethanol. Then hemolymph samples were collected by introducing a sterilized glass capillary pulled to a fine point between the second and third abdominal sternite. Subsequently, on average, 0.5 µl hemolymph were added to 15 µl of GM in a 0.5 ml Eppendorf tube on ice. Then females were dissected in GM and the mesospermalege removed and rinsed in 10 µl of sterile GM on a glass slide. After rinsing, the mesospermalege was put into a 0.5 ml Eppendorf tube containing 15 µl of sterile GM on ice. Using a sterile plastic pestle (Z359947, Sigma-Aldrich, Dorset, UK) and thorough vortexing, we homogenized the mesospermalege samples before spreading them on agar plates.

Each hemolymph and mesospermalege sample was split into two, i.e., 7 µl each, and spread with sterile glass beads (3 mm, Merck) on 1.5% LB agar plates (60 mm). We also ran procedural controls to check for potential contamination. Agar plates were incubated at 26°C until visible colonies were present, at most 48 h. After incubation, we screened for visible colonies and different colony morphotypes. These were picked and re-cultured to obtain single clonal cultures. From 74 samples, glycerol stocks were prepared and served as a culture collection for future analysis.

### DNA Extraction and PCR Amplification of the 16S rRNA Gene

**Figure 1:**
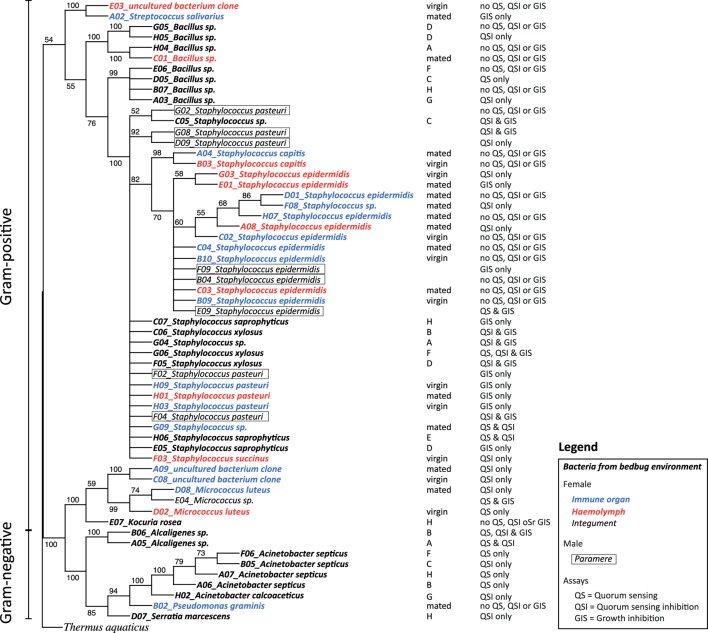
Phylogenetic tree [RAxML rapid bootstrap (35)], reconstructed for the 16s rDNA gene sequences from bacteria in bedbugs and their environment. Bacteria are given in different fonts depending on their location. Bacteria from filter papers on which bedbug stock populations were kept are given in bold italic, bacteria from female hemolymph samples are given in red italic, bacteria from female immune organs are given in blue italic, bacteria from female integuments are given in italic, and bacteria from parameres are given in italic and boxed. In the column next to the tree, the stock population ID and the mating status of females are presented, including the ability of the isolated bacteria to perform quorum sensing (QS), quorum sensing inhibition (QSI), or growth inhibition of the indicator strain (GIS).

Each glycerol stock was used to grow a liquid culture for extracting DNA (MO BIO, Ultra Clean, Microbial DNA Isolation Kit, Cat. Nr. 12224-250, Cambio Ltd., Cambridge, UK). DNA samples were sent to SourceBioscience Geneservice™ (Nottingham, UK) for PCR amplification and forward sequencing of a fragment of the 16s rRNA gene using the universal primer 27F (5′-AGAGTTTGATCMTGGCTCAG). The obtained sequences were trimmed at the 5′ and 3′ end to remove ambiguous parts, i.e., non-identified nucleotides, in order to optimize blast results and sequence alignment. Due to their poor quality, four sequences had to be excluded from the analysis. We used blast2go^1^ for the blasting of the sequences and ClustalX^2^ for the sequence alignment. The Maximum Likelihood phylogenetic tree of the bacterial sequences was determined by using the web interface RAxML Black Box^3^ (Figure 1) and NJplot to draw the phylogenetic tree^4^ (Figure 1).

### QS Assay

*N*-acyl homoserine lactone (AHL) production was measured as a surrogate for QS using the indicator strain *Chromobacterium violaceum* (CV026), which was assayed with all samples from our bacterial culture collection. CV026 does not produce C6-HSL but does produce violacein (purple pigment) in response to the presence of exogenous C6-HSL. This violacein production in strain CV026 is inducible by AHLs with N-acyl side chains from C4 to C8 in length. In contrast, AHLs with *N*-acyl side chains from C10 to C14 inhibit violacein production. Therefore, CV026 can be used as an indicator strain to detect a variety of AHLs.

*Chromobacterium violaceum* was first grown overnight in liquid LB broth. By mixing 50 ml of the overnight culture with 200 µl of warm 1% agar medium, we produced assay plates (10 ml in 90 mm Petri dishes). Then, we punched holes in the agar using the top end of a glass Pasteur pipette. For each sample from our bacteria stock library, two overnight cultures were produced (*N* = 148 overnight cultures). From each of those, we ran two replicates on two different plates. We added 50 µl of overnight culture to a hole in an assay plate. As a positive control, we also assayed the indicator strain *C. violaceum* ATTC 31532 that produces AHL. After inoculation with a bacteria sample, the plates were incubated for 48 h at 30°C. Then, each sample was scored for the occurrence of purple coloration around the well, which is the positive test of AHL production. No coloration indicated a negative test. For each positive assay, we measured the zone diameter twice in a perpendicular fashion and subsequently calculated the area of the zone in square millimeters.

### QSI Assay

Similar to the QS assay, we used a *C. violaceum* strain (ATTC 12472) as an indicator for QSI. In this strain, *N*-(3-hydroxydecanoyl)-l-homoserine lactone controls violacein production, a purple pigment, by QS (36). As the growth medium did not affect the QS assay, we produced assay plates in the same way as described above only with LB medium. Again, we tested all samples from our culture collection twice and with two overnight cultures. Plates were incubated for 48 h at 30°C. As the indicator strain is producing the purple pigment constantly when growing, a positive test of QSI is seen as a white, milky zone around the well. A negative test would be no zone and purple coloration right up to the edge of the well. In our case, we also observed clear zones around the well, which we scored as growth inhibition of the indicator strain (GIS). For each positive assay (QSI and GIS), we measured the zone diameter twice in a perpendicular fashion and subsequently calculated the area of the zone in square millimeters.

### Statistical Analysis

(i) *Site-specific, culturable microbiome of the bedbug*: the number of culturable bacteria was similar on the six growth media used (Table 2). We thus pooled the data to identify differences between numbers of species found in different organs and mating status with a Chi-squared test. We compared the number of females from which bacteria could be cultivated versus the number of females from which no bacteria could be cultivated using a Fisher Exact test.
(ii) *The effect of wounding and feeding on the microbiome of female bedbugs*: due to the low number of different bacteria species found in the differently treated females, we did not perform a statistical analysis (Table 3). As the sample sizes were limited, we fitted Fisher Exact tests to identify differences between numbers of females from which bacteria could be cultivated in comparison to females from which no bacteria could be cultivated with either mating or feeding status as a factor.

Quorum sensing, QSI, and GIS were analyzed in a qualitative manner by giving an account of how many culturable bacteria species were able to perform QS, QSI, or GIS in relation to mating status, bedbug population, and tissue. All statistical analyses were conducted using R 3.4.1 (37).

## Results

### Overall Diversity of Species and of Quorum Communication

In total, we identified 20 different culturable bacterial species across all our samples (five Gram-negative, thirteen Gram-positive bacteria, and two clones that were not identified) (Table 1; Figure 1). Ten species were cultured from the environment of the bedbug (four Gram-negative, six Gram-positive bacteria), eleven from female tissues, and two from males (Table 1). Samples from the same bacteria species that were sampled from different collection sites (tissues) clustered together in the phylogenetic analysis (Figure 1).

**Table 1:** Bacterial species found in bedbugs and their environment for all experiments combined.

Female								Male	
Bedbug environment	*N* stocks	Integument	*N*	Hemolymph	*N*	Immune organ	*N*	Paramere	*N*
*Alcaligenes* sp.^G−^	2	*Micrococcus* sp.	1	*Bacillus* sp.	0;1	*Pseudomonas graminis*^G−^	0;1	*Staphylococcus epidermidis*	3
*Acinetobacter septicus*^G−^	4	*Staphylococcus pasteuri*	1	*Micrococcus luteus*	1;0	*M. luteus*	0;1	*Staphylococcus pasteuri*	5
*Acinetobacter calcoaceticus*^G−^	1			*Staphylococcus capitis*	1;0	*S. capitis*	0;1		
*Serratia marcescens*^G−^	1			*S. epidermidis*	1;3	*S. epidermidis*	3;3		
*Bacillus* sp.	6			*Staphylococcus pasteuri*	0;1	*S. pasteuri*	2;0		
*Kocuria rosea*	1			*Staphylococcus succinus*	1;0	*Staphylococcus* sp. *(2)*	0;1		
*Staphylococcus sp. (1)*	1			Unidentified bacterial clone 1	1;0	*Streptococcus salivarius*	0;1		
*Staphylococcus sp. (3)*	1					Unidentified bacterial clone 2	1;1		
*Staphylococcus saprophyticus*	3								
*Staphylococcus xylosus*	3								
Number of units/individuals screened	16		3		13;20		13;20		12

*For the environment, two samples from eight stock populations were screened. Hemolymph and immune organs were sampled in virgin and mated females from only one stock population. For these, the number of samples with bacteria was given for both mating states (virgin;mated). The bottom row gives the number of units or individuals screened*.

*G^−^, Gram-negative bacterium*.

**Figure 2:**
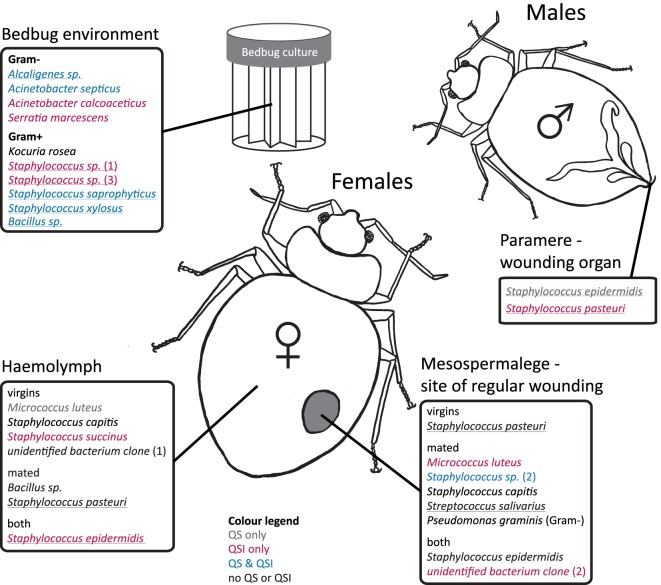
Distribution of cultivated bacteria with respect to female mating status, bedbug tissue, and bedbug environment. Species names are colored according to their ability to perform quorum sensing (QS) or quorums sensing inhibition (QSI). Underlined species names indicate the ability to perform growth inhibition (GIS) of the indicator strain. Cultivated bacteria that did not show any reaction to the indicator strains in the assay are given in black.

**Figure 3:**
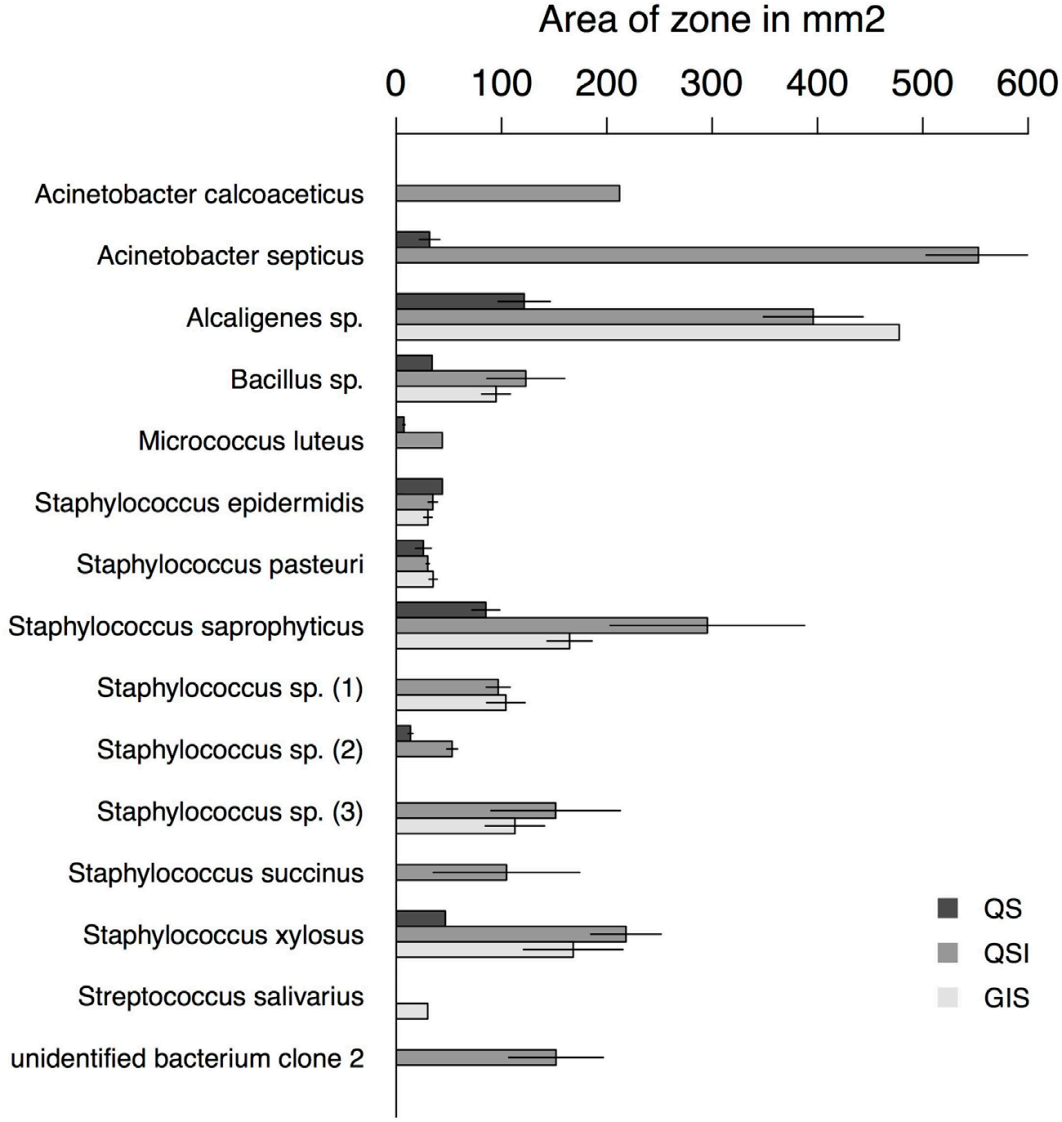
Area of zones in square millimeters for each assay type and bacteria species with at least one positive assay. Error bars represent the SE.

Overall, 56% of the cultivated bacteria isolates showed QS (Gram-negative bacteria: 40%; Gram-positive bacteria: 67%), 72% showed QSI (Gram-negative bacteria: 80%; Gram-positive bacteria: 67%), and 50% showed growth inhibition of the indicator strain (GIS) (Gram-negative bacteria: 20%; Gram-positive bacteria: 67%). Generally, QSI response was strongest showing a mean area of 175.98 mm ± 151.13 mm (mean ± SD), GIS zones were on average 135.20 mm ± 138.97 mm, and QS areas 45.50 mm ± 36.30 mm. The expression of QS, QSI, and GIS of the different bacteria isolates was dependent also on geographic origin, mating status and tissue (Figure 2). For example, eight of the nine bacteria species found in the environment showed QS, QSI, or GIS or a combination of those three (Figure 2) but only one of the bacteria species cultured from female tissues performed both QS and QSI. The strength of response in QS, QSI, and GIS varied between species (Figure 3). From five of the six field-caught bedbug populations, environmental bacteria could be cultivated that were able to quorum sense and/or to inhibit the growth of the indicator strain (Figure 1). Of the lab populations, one harbored bacteria that could perform QSI, but not QS. Environmental bacteria grown from the second laboratory population could perform QS, QSI, and GIS. All bedbug populations had cultivable bacteria that were able to quorum quench the signal of the indicator strain (Figure 1).

In total, we recovered 24 isolates from the bedbug environments of the eight stock populations (UK: 14, Kenya: 3, and Lab: 7). In the environment of the UK bedbug stock populations, we found seven, in the Kenyan population three and in the Lab stocks six different bacteria species. Five bacteria species were only found in one population [A: *Staphylococcus* sp. (1); C: *Staphylococcus* sp. (3); G: *Acinetobacter calcoaceticus*; H: *Kociura rosea* and *Serratia marcescens*] (Figure 1). In three UK populations (A, B, C), in the Kenyan population and in one Lab stock (G) bacteria showed QS, QSI, and GIS. One UK population contained bacteria that only showed QSI and GIS (D) and another only QS and QSI (E). And the second Lab stock only had bacteria showing QSI (Figure 1).

#### Site-Specific, Culturable Microbiome of the Bedbug

##### Species

In the hemolymph of a mated female, we found a *Bacillus* sp. From the hemolymph of virgin females, we cultivated *Micrococcus luteus*. The mesospermaleges of a mated and a virgin female yielded an unidentified bacterium clone (2) that clustered with *Micrococcus* sp. (Figure 1). In addition to *M. luteus*, the mesospermaleges of mated females harbored *Streptococcus salivarius* and the Gram-negative *Pseudomonas graminis* (Figure 2). Parameres—the male wounding organs—harbored *Staphylococcus epidermidis* and *Staphylococcus pasteuri* (Figure 2). One female harbored four different types of bacteria (hemolymph: *S. epidermidis* and *S. pasteuri*; mesospermalege: *Staphylococcus* sp. and *S. salivarius*).

**Figure 4:**
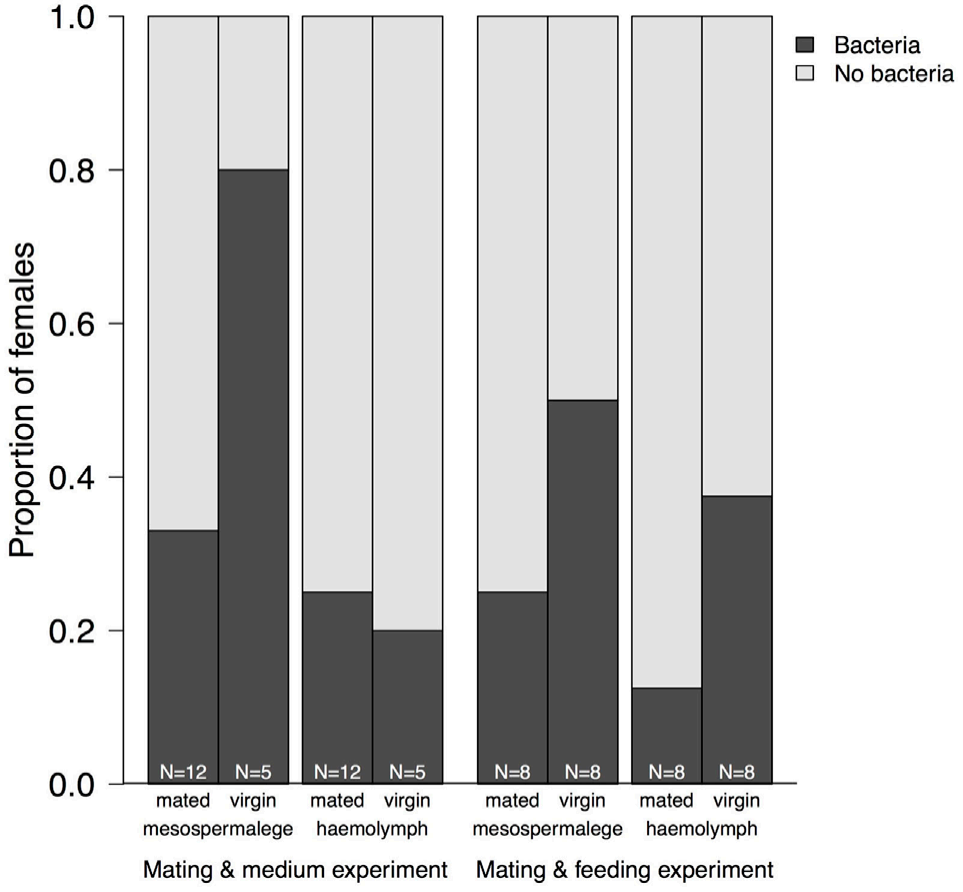
Proportion of mesospermaleges—the site of regular wounding—from mated and virgin females from which bacteria could be cultivated and their hemolymph in contrast to the number of females without bacteria. The sample sizes below indicate the number of females screened for bacteria. “Mating and medium experiment” refers to the screen of the site-specific, cultivable microbiome of the bedbug and “Mating and feeding experiment” refers to the test of the effect of wounding and feeding on the microbiome of female bedbugs.

Whereas after mating, the number of bacteria species increased in comparison to virgin bedbugs, the proportion of females harboring bacteria decreased after mating (Figure 3; Table 3). From the hemolymph of virgin females, one bacteria species could be grown, but four from the hemolymph of mated females (Table 3). Mating status and tissues did not differ in the number of females from which bacteria could be grown (Fisher’s Exact test: *P* = 0.19). Four of twelve mesospermaleges (33%) of mated females contained cultivable bacteria, in contrast to four of five mesospermaleges (80%) of virgin females, a difference that was, however, not significant (Fisher’s Exact test: *P* = 0.13) (Table 3). Different growth media did not affect the number (Fisher’s Exact test: *P* = 1) or type of bacteria species that could be cultivated (Table 3). Although not significant (Fisher’s Exact test: *P* = 0.15), in both experiments, mating reduced the number of culturable bacteria found in the mesospermalege—the site of regular wounding (Figure 4). Overall, female hemolymph showed a lower proportion of cultivable bacteria than the mesospermalege—the site of regular wounding (Figure 4). Mesospermaleges of mated females contained less than half as many cultivatable bacteria as the same organs from virgin females and the hemolymph of showed a similar pattern (Figure 4).

##### Quorum Communication

Bacteria from female tissues seemed to be limited to one form of communication (e.g., only QS in *M. luteus* from hemolymph of virgin female; only QSI in *Staphylococcus* sp. from hemolymph of virgin female; or only GIS in *S. pasteuri* from hemolymph of mated female) (Figure 2). Three bacteria species from mesospermaleges of mated females showed QSI (Figure 2), one of which was the same in mesospermaleges of virgin females. *S. salivarius* from mated and *S. pasteuri* from virgin female mesospermaleges showed GIS and the hemolymph of mated females contained no bacteria showing either QS or QSI, but two performed GIS. In contrast, the hemolymph of virgin females harbored one cultivable bacteria species with QS, one with QS but none with GIS (Figure 2). *M. luteus* from the mesospermalege of mated females perform QSI, whereas the *M. luteus* found in virgin hemolymph were only able to QS. We found a similar contrast for parameres and female tissues. *S. epidermidis* from a paramere performed QS and GIS, *S. epidermidis* found in the hemolymph of females performed QSI and GIS (Figure 2). While *S. pasteuri* from a paramere could quench and inhibit the indicator strain, *S. pasteuri* from females would only inhibit the growth of it.

#### The Effect of Wounding and Feeding on the Microbiome of Female Bedbugs

##### Species

In addition to the eight bacteria species found while investigating the site-specific, culturable microbiome of the bedbug, we identified another three when testing for the effect of wounding and feeding on the microbiome of female bedbugs. The proportion of females from which bacteria were cultivated was dependent on the females’ mating and feeding status (Table 3; Fisher’s Exact test: *P* = 0.04).

Mated females harbored a lower number of culturable bacteria species than virgin females (Table 3). In addition, fewer mated females harbored bacteria than virgin females (Fisher’s Exact test: *P* = 0.12) (Figure 4). Mated fed females harbored bacteria whereas we could not grow bacteria from unfed mated females (Fisher’s Exact test: *P* = 0.14). From mated fed females, we identified *S. epidermidis, S. capitis*, and one *Staphylococcus* sp. From four fed and three unfed virgin females, we could cultivate bacteria (Table 2). The proportion of fed and unfed females from which bacteria could be cultivated did not differ (Fisher’s Exact test: *P* = 0.12) (Table 3). Virgin fed females harbored *S. capitis, S. succinus*, one *Staphylococcus* sp. and one unidentified bacterium clone (1) similar to *S. salivarius* (Figure 1). The *S. succinus* and the unidentified bacterium clone (1) originated from the same female. From two unfed virgin females, we identified *S. epidermidis* and from one *S. pasteuri*. And from the washed female integuments, we cultivated *S. pasteuri* and a *Micrococcus* sp. Two of the seven screened males harbored two bacteria species, *S. epidermidis* and *S. pasteuri*, on their paramere (Figure 2). These two bacteria species were most frequently found in both experiments (site-specific, culturable microbiome of the bedbug: *S. epidermidis*: 2 parameres, 2 hemolymph samples, 4 mesospermaleges, *S. pasteuri*: 3 parameres, 1 hemolymph sample, 1 mesospermalege; effect of wounding and feeding on the microbiome of female bedbugs: *S. epidermidis*: 1 paramere, 2 hemolymph samples, 2 mesospermaleges, *S. pasteuri*: 2 parameres, and 1 mesospermalege).

**Table 2:** Effect of mating and test medium on bacteria presence in female bedbugs.

Treatment	*N*	Growth medium						Number of different bacteria species	Percent females or samples with bacteria
		LB	GM	NB	NBTA	Potato extract	R-2A		
Mated	12	4	5	4	4	4	4	7	42
Hemolymph		2	2	2	2	2	3	4	25
Mesospermalege		2	3	2	2	2	1	4	33
Virgin	5	3	2	3	2	2	2	3	80
Hemolymph		1	0	0	0	0	0	1	20
Mesospermalege		2	2	3	2	2	2	3	80

*Numbers given for growth media are the number of different bacteria species found in each tissue*.

**Table 3:** Effect of mating and feeding on bacteria presence in tissues from female bedbugs.

Treatment	*N*	Mean mating duration in seconds (±SD)	Percent females with bacteria	Number of different bacteria species found
**Fed**				
Mated	4	99.0 ± 9.7	75	2
Virgin	4		100	4^a^
**Unfed**				
Mated	4	80.5 ± 22.3	0	0
Virgin	4		75	2

*^a^Two different bacteria species from the same individual*.

##### Quorum Communication

One bacterium from mated females showed QSI in contrast to three from virgin females. Virgin females also harbored bacteria capable of GIS. Fed females harbored three bacteria species that showed QSI and three that did not signal. From unfed females, we recovered bacteria that were able of QSI and GIS. None of the bacteria found in this experiment showed QS.

## Discussion

We advocate the bedbug mating as a suitable system to study the effects of bacteria on reproduction and to address prokaryote and eukaryote communication during wounding in a natural system. We found that most organs of bedbugs harbor bacteria, which are capable of both signaling pathways, QS and QSI. Some of the bacteria were able to stop the growth of an indicator strain indicating potential higher competitiveness. Finally, we show that the microbiome varies between mated and non-mated individuals, between fed and non-fed ones as well as between organs. By focusing on the prokaryotic quorum communication system, we provide a baseline for future research in this unique system.

### The Bedbug Microbiome

In 2013, the first in-depth assessment and characterization of the bed bug (*Cimex lectularius*) microbiome was conducted (38). Although variation in diversity and structure was found among geographical locations, the presence of similar bacterial lineages across populations provided evidence for the presence of a *Cimex* core microbiome. To date, several studies including our own have found similar bacterial taxa in bedbug populations from all over the world (31, 38, 39) further supporting the existence of a core microbiome. However, our study also shows considerable variation in the bedbug microbiomes of different tissues, sexes, and reproductive states. In addition, bacteria found in the bedbug environment differed from the ones found in female or on male reproductive organs, hinting at a specific interaction between bacteria and host. Isolates from the same bacterial species that were sampled from different collection sites (tissues) clustered together in the phylogenetic analysis. These bacteria species seem to rather opportunistically colonize tissues and do not select a habitat within hosts. It is, therefore, likely that those bacterial species get transmitted between individuals regularly.

Any bacterium specifically associated with only one organ, i.e., similar to an endosymbiont, might benefit from using QS and QSI to occupy the niche and protect the host organ from intruding bacteria. Most bacteria found in the bedbug environment performed QS, QSI, or GIS in some combination, except for three species. Only one did not perform any QS, QSI, or GI. In contrast, none of the bacteria from male or female bedbugs was performing QS, QSI, or GIS in combination, suggesting that either the host suppresses signaling or no need was present for the bacteria to communicate. Whereas several bacteria species in mated females showed QSI or GIS when isolated from the mesospermalege (the site of regular wounding), only one bacterium showed QS. Whether this indicates a certain degree of specificity of the given bacteria in this tissue remains to be shown.

While organ-specific bacterial communities are well established for humans [e.g., Ref. (1)], whether quorum communication can also be organ-specific seems less clear. Currently, evidence is lacking for such a specificity. But given the intricate interaction and communication between animals and bacteria already described (40), an organ-specific communication would not be unlikely. We found no previous report on quorum communication of bacteria associated with bedbugs. In our study, we not only identified a diverse range of QS and QSI communicating bacterial species but we also found that some aspects appeared to be related to the organ from which the culturables were isolated. For example, we found that *M. luteus* showed QSI when sampled from the mesospermalege but not when sampled from the hemolymph. Similarly, *S. epidermidis* showed QS and GIS on the paramere, QSI and GIS in the hemolymph, and no QS, QSI, or GIS in the mesospermalege. Although we examined QS and QSI *in vitro*, it is unlikely that these differences arose from the *in vitro* conditions, because we treated all culturables the same way. At least some aspect correlated to eliciting a QS or QSI response *in vitro* must have been different between the organs.

### Sex Differences and the Effect of Mating on the Microbiome and Its Quorum Communication

The sex differences in the microbiome of bedbugs that we found agree with many other species and are not surprising, given the large habitat difference the male and female reproductive organs represent to microbes. For example, mosquitoes, mice, and humans show differences in microbiome diversity or abundance of bacteria taxa between the sexes (41–43). Sex differences in the microbial community can even lead to sex-specific hormone regulation in mice (42), suggesting differences in communication between the microbiome and its host. However, habitat differences [e.g., Ref. (15, 44)] and sexual transmission are not the only determinants of sex differences in the microbial community of animal genitalia. For example, Gendrin et al. (45) showed that the deposition of infectious microbes on the genital plate caused a systemic, rather than a localized immune response in males but not in females.

Mated females had fewer bacterial species in the mesospermalege than virgin females, a trend that was also found in the hemolymph. This observation implies that the site of regular wounding—the mesospermalege—has a role in controlling bacteria transmission or bacteria growth. This is consistent with the finding that in bedbug females, the growth inhibition of bacteria and antibacterial activity of the mesospermalege is stronger in mated than virgin females (O. Otti, unpublished data). It is, therefore, possible that part of the observed bacterial reduction in the mesospermalege of mated females might be caused by an upregulation of the growth inhibition factors during and/or the production of constitutive immune agents, such as lysozyme, during or even in anticipation of mating [see Ref. (19) for an example in *Drosophila*].

None of the bacteria recovered from mated females showed QS. Actually, QS was only found in one bacterial clone from the hemolymph of a virgin. Bacteria from the hemolymph of mated females did neither quorum sense nor quench, whereas bacteria from the hemolymph of virgin females were capable of both signaling pathways. Females might control the signaling or growth of bacteria in the hemolymph during reproduction to minimize the risk of a systemic infection. Although the investment into pathogen protection is often reduced *via* immune suppression during reproduction (46), regular wounding combined with a threat of genital infection might select for means to control and localize bacterial growth.

### The Effect of (Blood) Feeding on the Microbiome and Its Quorum Communication

Feeding increased the number of species collected from female bedbugs. Unfed mated females were even free from culturable bacteria. Only two species were collected from unfed females. Interestingly, bacteria from fed females only showed QSI or no signaling at all, whereas unfed females were capable of QSI and GIS. The reason for these differences will have to be investigated further.

## Conclusion

We characterize the culturable microbiome for a system with natural regular sexual wounding and show that the host’s sex as well as feeding and mating, are associated with striking differences in the microbiome and the quorum communication system. Despite the uniqueness of the system, the frequency of copulatory wounding suggests that similar differences might be worth studying in other eukaryotic hosts and may represent an important part of the metaorganism.

## Author Contributions

OO, PD, KH, and KR conceived the idea and designed the experiment; performed the statistical analysis; and interpreted the results and wrote the manuscript. OO and PD carried out the experiment. All authors read and approved of the final manuscript.

## Conflict of Interest Statement

The authors declare that the research was conducted in the absence of any commercial or financial relationships that could be construed as a potential conflict of interest.

## Footnotes

^1^http://www.blast2go.com.

^2^http://www.clustal.org/clustal2/.

^3^https://embnet.vital-it.ch/raxml-bb/

^4^http://pbil.univ-lyon1.fr/software/njplot.html.
